# Supplementary material for: Analysis of cancer mutations introduced into the Drosophila melanogaster Notch negative regulatory region uncovers a diversity of regulatory outcomes
Source: eLife. 2026 Aug 4;14:RP108812. doi: 10.7554/eLife.108812 (PMC13436963; doi:10.7554/eLife.108812)

Figure 6, Source data 2. Original western blots corresponding to Figure 6 panel A and additional repeats used to quantify data in panel B

1. no Notch
2. WT
3. E1489V
4. D1497N
5. A1504V
6. G1515K
7. T1523M
8. N1529E
9. E1538R
10. D1548N
11. E1553P
12. R1554C
13. L1562P

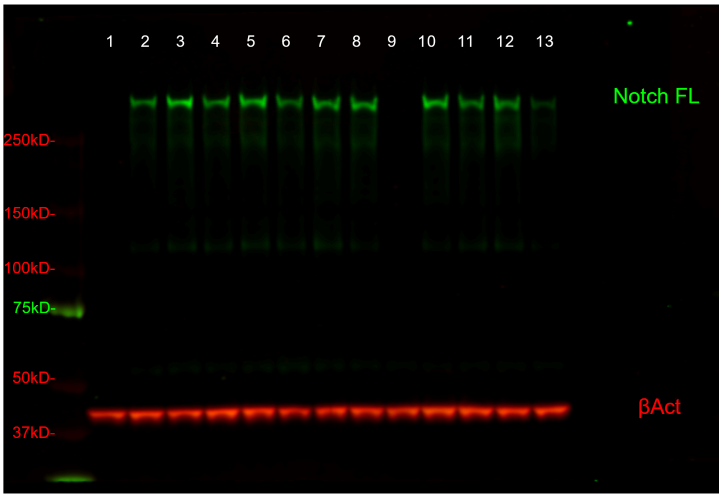

14. H1570P
15. D1573V
16. Y1578R
17. F1617P
18. R1626Q
19. H1630P
20. L1632P
21. R1633P
22. L1686P
23. E1705P
24. ΔPEST
25. WT
26. no Notch

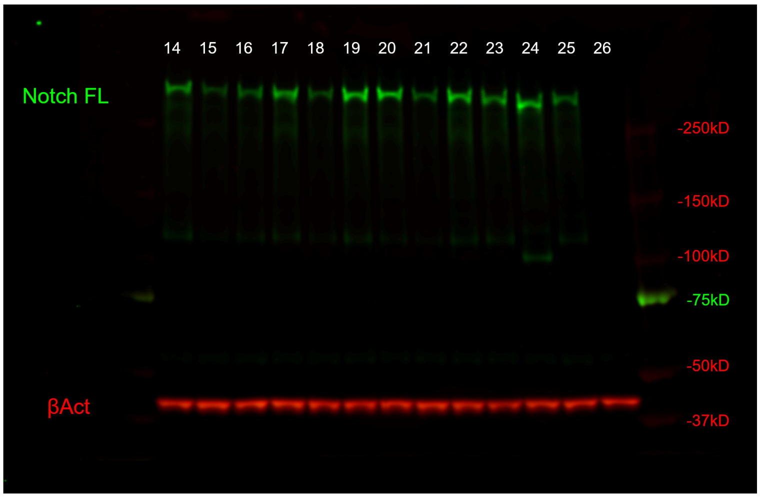

1. no Notch
2. WT
3. E1489V
4. D1497N
5. A1504V
6. G1515K
7. T1523M
8. N1529E
9. E1538R
10. D1548N
11. E1553P
12. R1554C
13. L1562P

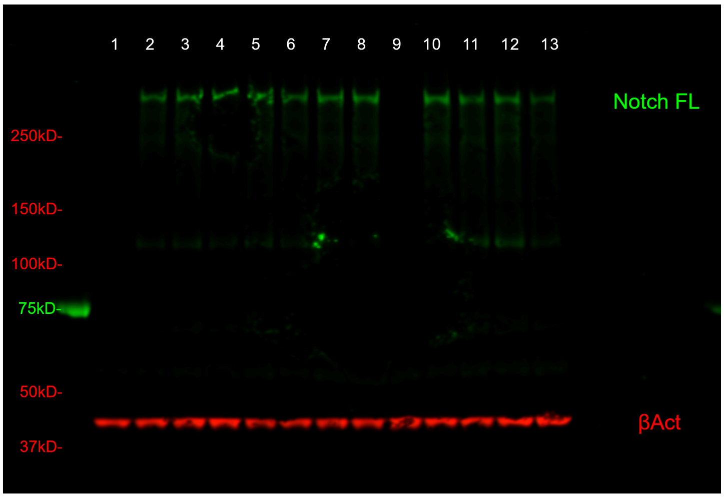

14. H1570P
15. D1573V
16. Y1578R
17. F1617P
18. R1626Q
19. H1630P
20. L1632P
21. R1633P
22. L1686P
23. E1705P
24. ΔPEST
25. WT
26. no Notch

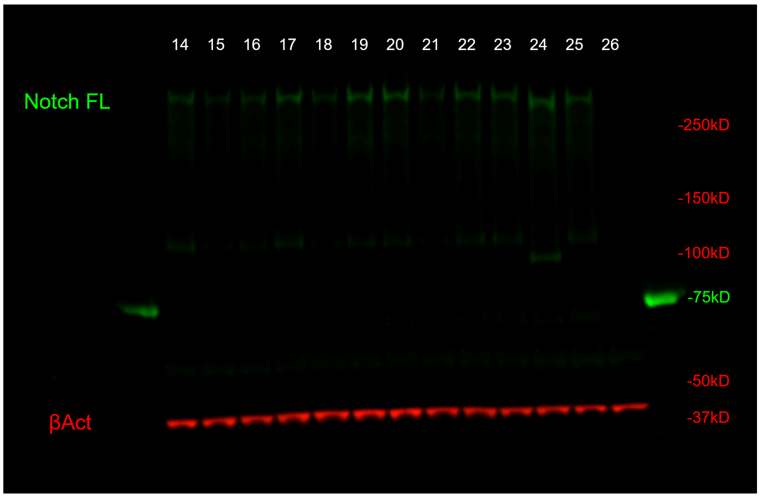

1. no Notch
2. WT
3. E1489V
4. D1497N
5. A1504V
6. G1515K
7. T1523M
8. N1529E
9. E1538R
10. D1548N
11. E1553P
12. R1554C
13. L1562P

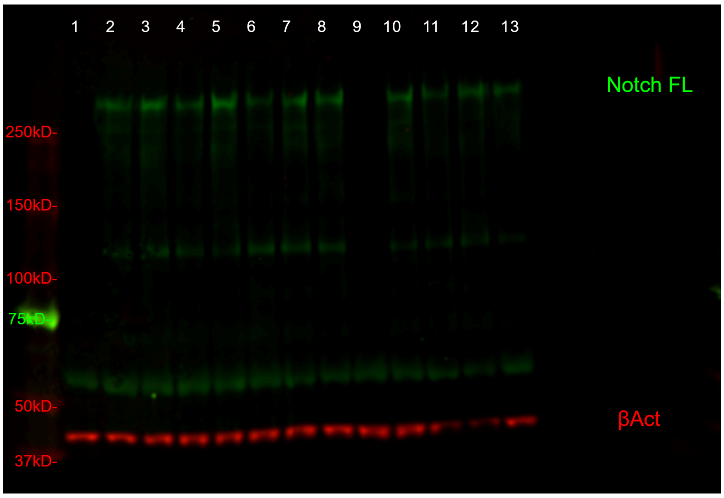

14. H1570P
15. D1573V
16. Y1578R
17. F1617P
18. R1626Q
19. H1630P
20. L1632P
21. R1633P
22. L1686P
23. E1705P
24. ΔPEST
25. WT
26. no Notch

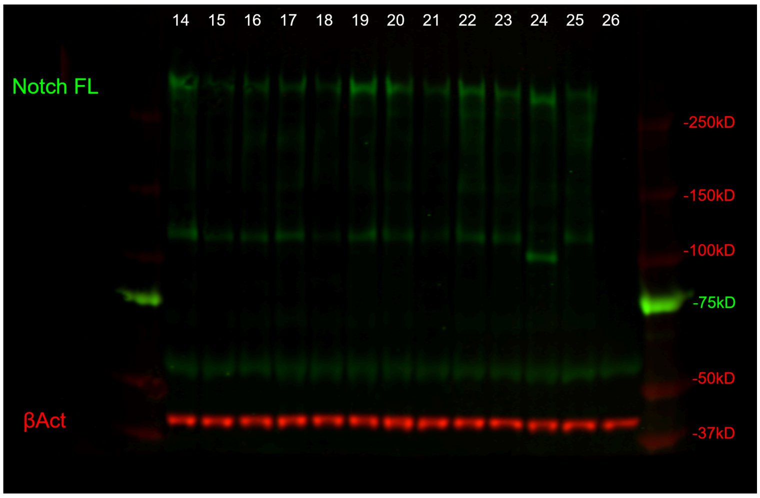

Supplement: Supplementary file 3 [file elife-108812-fig6-data3.zip › Figure 6 Source data 3/Figure 6 A,B Source Data 3.pdf]
