# Supplementary material for: Analysis of cancer mutations introduced into the Drosophila melanogaster Notch negative regulatory region uncovers a diversity of regulatory outcomes
Source: eLife. 2026 Aug 4;14:RP108812. doi: 10.7554/eLife.108812 (PMC13436963; doi:10.7554/eLife.108812)
Supplement: Supplementary file 3 [file elife-108812-fig6-data3.zip › Figure 6 Source data 3/Figure 6 C,D Source data 5.pdf]

Figure 6, Source data 3. Original western blots corresponding to panel C and additional repeats used to quantify data in panel D.

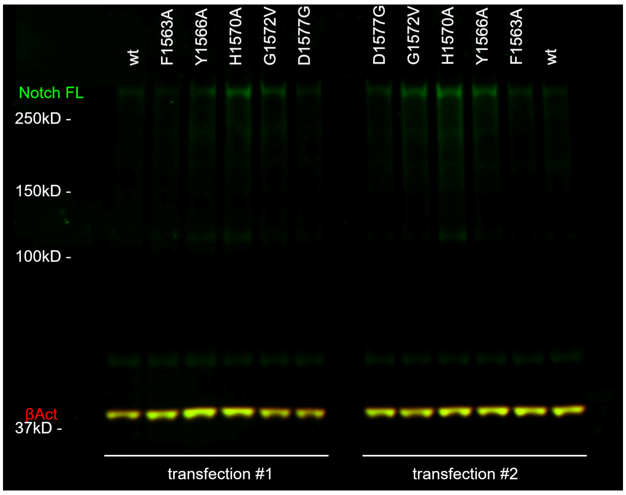

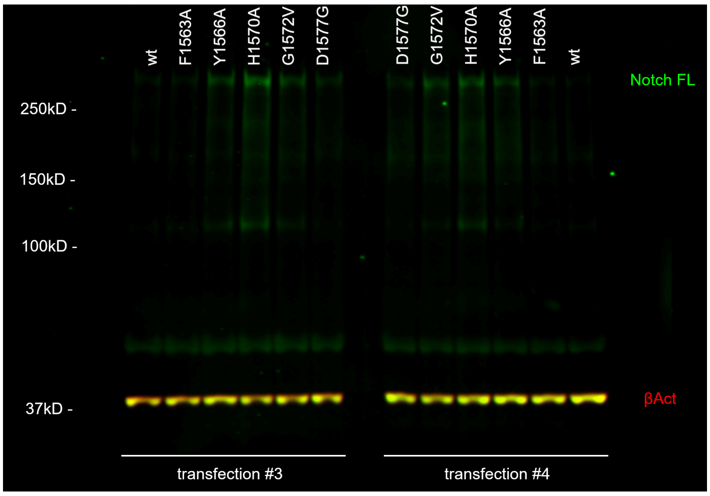
wt
F1563A
Y1566A
H1570A
G1572V
D1577G
D1577G
G1572V
H1570A
Y1566A
F1563A
wt
Notch FL
250kD -
150kD -
100kD -
βAct
37kD -
transfection #3
transfection #4
