# Supplementary material for: Analysis of cancer mutations introduced into the Drosophila melanogaster Notch negative regulatory region uncovers a diversity of regulatory outcomes
Source: eLife. 2026 Aug 4;14:RP108812. doi: 10.7554/eLife.108812 (PMC13436963; doi:10.7554/eLife.108812)
Supplement: Supplementary file 3 [file elife-108812-fig6-data3.zip › Figure 6 Source data 3/Figure 6 E,F Source data 7.pdf]

Figure 6, Source data 4. Original western blots used for panel E and additional repeats used for quantification in panel F

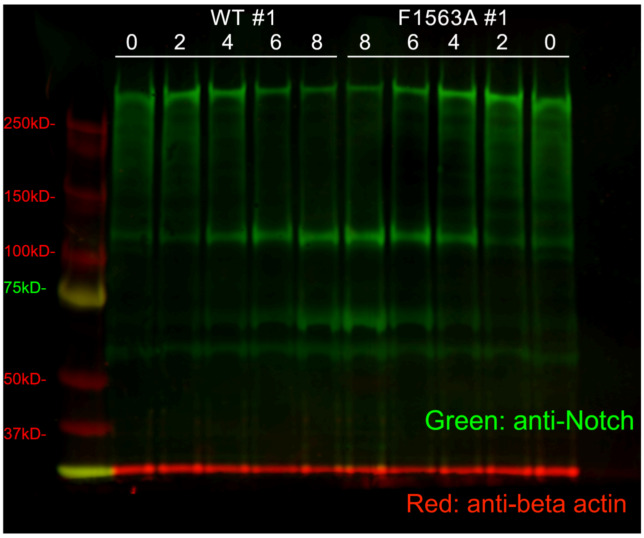

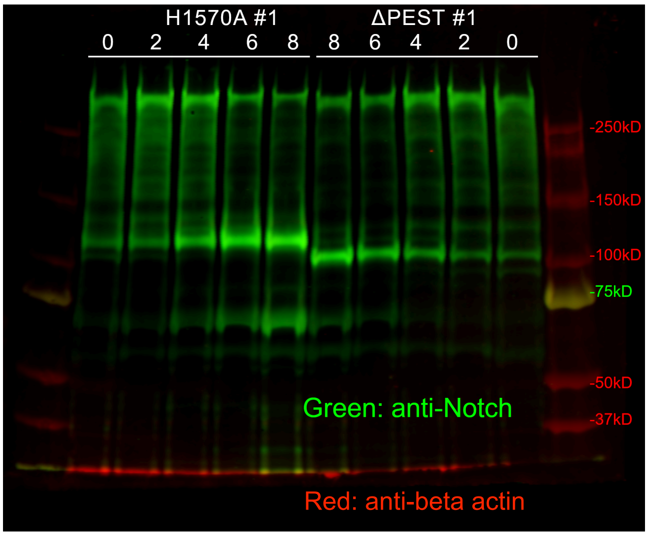
H1570A #1
0 2 4 6 8
ΔPEST #1
8 6 4 2 0
-250kD
-150kD
-100kD
-75kD
-50kD
-37kD
Green: anti-Notch
Red: anti-beta actin

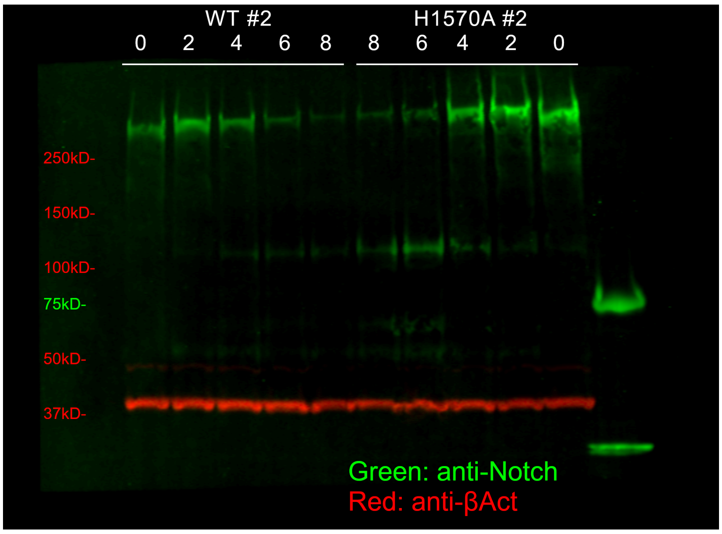
WT #2
H1570A #2
0 2 4 6 8
8 6 4 2 0
250kD-
150kD-
100kD-
75kD-
50kD-
37kD-
Green: anti-Notch
Red: anti-βAct

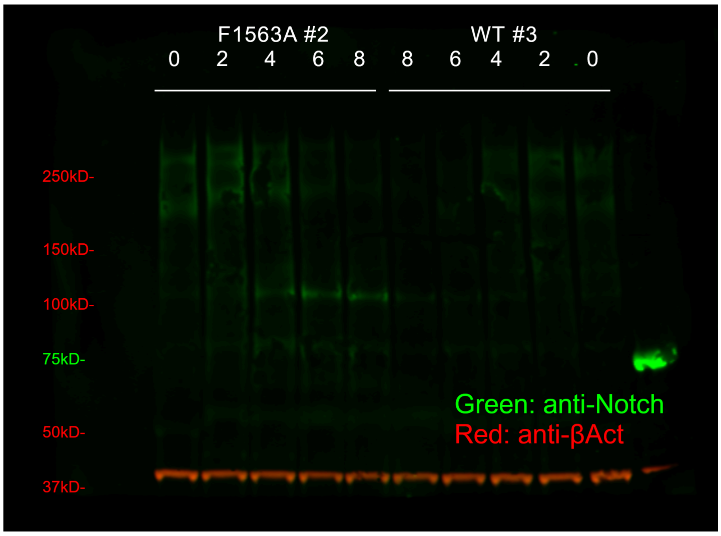
F1563A #2
0 2 4 6 8
WT #3
8 6 4 2 0
250kD-
150kD-
100kD-
75kD-
50kD-
37kD-
Green: anti-Notch
Red: anti-βAct

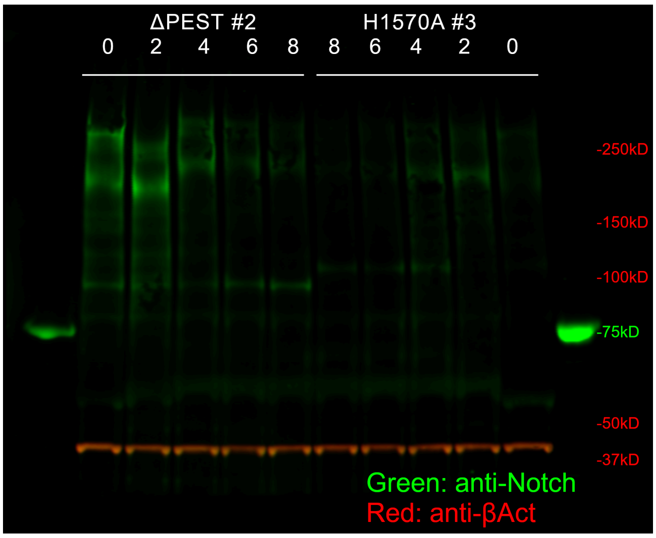
ΔPEST #2
0 2 4 6 8
H1570A #3
8 6 4 2 0
-250kD
-150kD
-100kD
-75kD
-50kD
-37kD
Green: anti-Notch
Red: anti-βAct

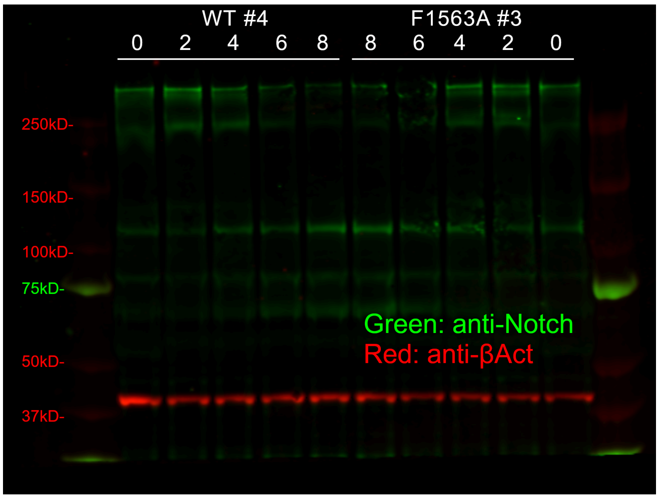
WT #4
0 2 4 6 8
F1563A #3
8 6 4 2 0
250kD-
150kD-
100kD-
75kD-
50kD-
37kD-
Green: anti-Notch
Red: anti-βAct

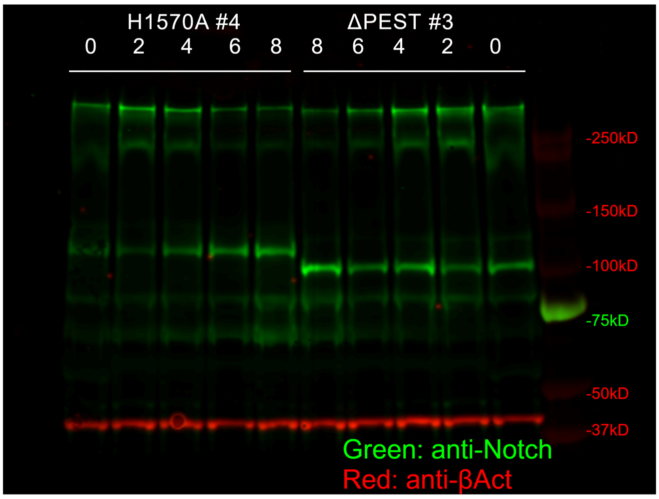
H1570A #4
ΔPEST #3
0 2 4 6 8 8 6 4 2 0
-250kD
-150kD
-100kD
-75kD
-50kD
-37kD
Green: anti-Notch
Red: anti-βAct
