# Supplementary material for: Analysis of cancer mutations introduced into the Drosophila melanogaster Notch negative regulatory region uncovers a diversity of regulatory outcomes
Source: eLife. 2026 Aug 4;14:RP108812. doi: 10.7554/eLife.108812 (PMC13436963; doi:10.7554/eLife.108812)
Supplement: Supplementary file 5 [file elife-108812-fig6-figsupp1-data3.zip › Figure 6 Supplement 1 Source data 3/Figure 6 supplement 1 Source data 3 .pdf]

Figure 6, supplement 1, Source data 2. Original western blots corresponding to panel A and additional repeats used to quantify data in panel B.

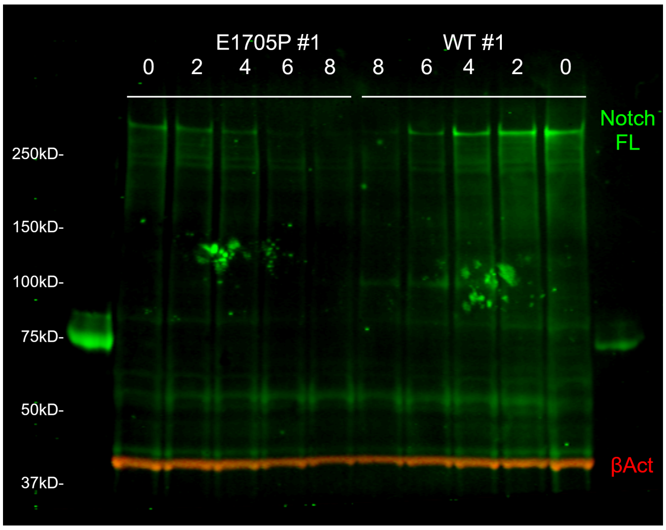

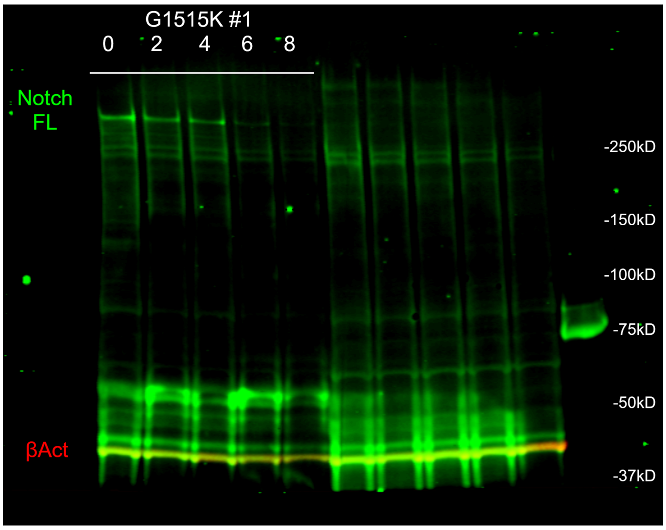
G1515K #1
0 2 4 6 8
Notch
FL
βAct
-250kD
-150kD
-100kD
-75kD
-50kD
-37kD

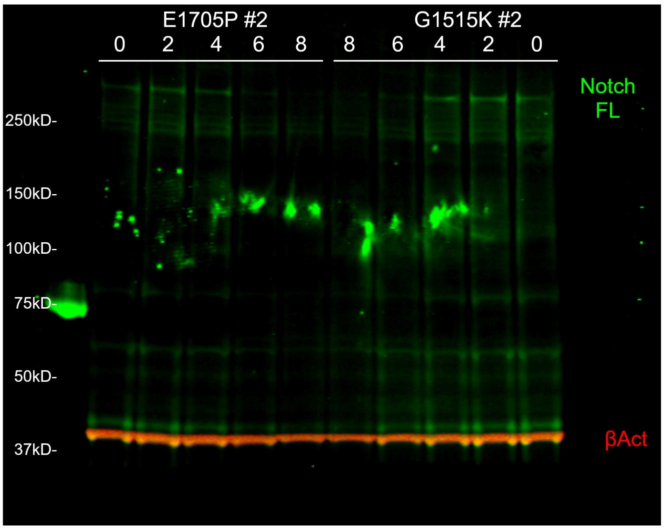
E1705P #2
G1515K #2
0 2 4 6 8
8 6 4 2 0
Notch FL
250kD-
150kD-
100kD-
75kD-
50kD-
37kD-
βAct

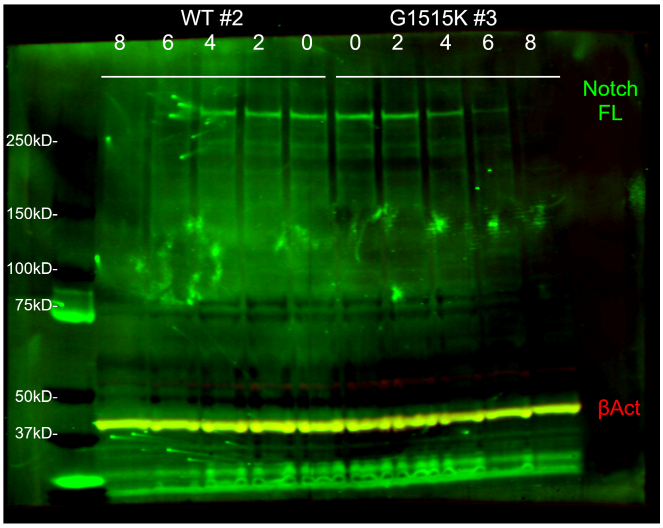

WT #2
G1515K #3
8
6
4
2
0
0
2
4
6
8
Notch FL
250kD-
150kD-
100kD-
75kD-
50kD-
37kD-
βAct

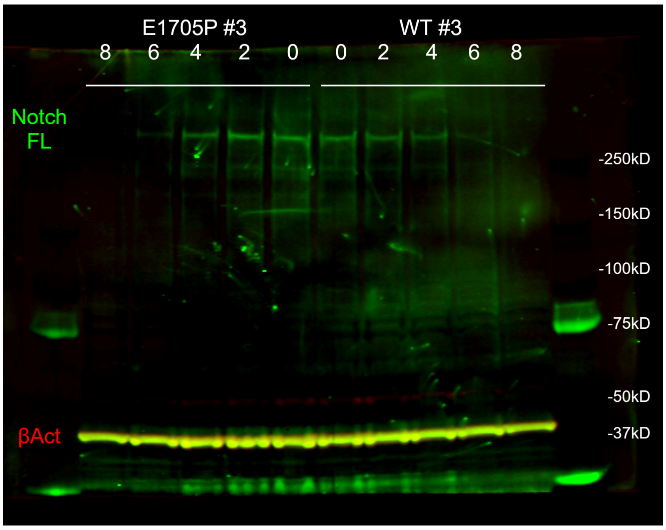
E1705P #3
WT #3
8 6 4 2 0 0 2 4 6 8
Notch FL
βAct
-250kD
-150kD
-100kD
-75kD
-50kD
-37kD
