# Supplementary material for: Analysis of cancer mutations introduced into the Drosophila melanogaster Notch negative regulatory region uncovers a diversity of regulatory outcomes
Source: eLife. 2026 Aug 4;14:RP108812. doi: 10.7554/eLife.108812 (PMC13436963; doi:10.7554/eLife.108812)
Supplement: Peer review history

# Editor report: eLife Assessment

**DOI:** 10.7554/eLife.108812.3.sa0

**Authors:** Apurva Sarin

**Affiliations:** Institute for Stem Cell Science and Regenerative Medicine India

**Keywords:** Convincing, Important

This **important** study presents a technically rigorous and carefully controlled analysis of the signalling potential of cancer-associated gain-of-function Notch alleles. The work is clearly presented, and the experiments are robust, comprehensive, and well-controlled. While some data primarily establish the system or report negative findings, the comparative approach in a well-characterized model provides **convincing** mechanistic evidence for how these Notch variants function. This study will be of interest to researchers in both developmental and cancer biology.

# Referee report: Reviewer #1 (Public review)

**DOI:** 10.7554/eLife.108812.3.sa1

Summary:

In their paper, Shimizu and Baron describe the signaling potential of cancer gain-of-function Notch alleles using the Drosophila Notch transfected in S2 cells. These cells do not express Notch or the ligand Dl or Dx, which are all transfected. With this simple cellular system, the authors have previously shown that it is possible to measure Notch signaling levels by using a reporter for the 3 main types of signaling outputs, basal signaling, ligand-induced signaling and ligand-independent signaling regulated by deltex. The authors proceed to test 22 cancer mutations for the above-mentioned 3 outputs. The mutation is considered a cluster in the negative regulatory region (NRR) that is composed of 3 LNR repeats wrapping around the HD domain. This arrangement shields the S2 cleavage site that starts the activation reaction.

The main findings are:

(1) Figure 1: the cell system can recapture ectopic activation of 3 existing Drosophila alleles validated in vivo.

(2) Figure 2: Some of the HD mutants do show ectopic activation that is not induced by Dl or Dx, arguing that these mutations fully expose the S2 site. Some of the HD mutants do not show ectopic activation in this system, a fact that is suggested to be related to retention in the secretory pathway.

(3) Figure 3: Some of the LNR mutants do show ectopic activation that is induced by Dl or Dx, arguing that these might partially expose the S2 site.

(4) Figure 4-6: 3 sites of the LNR3 on the surface that are involved in receptor heterodimerisation, if mutated to A, are found to cause ectopic activation that is induced by Dl or Dx. This is not due to changes in their dimerisation ability, and these mutants are found to be expressed at a higher level than WT, possibly due to decreased levels of protein degradation.

Strengths and Weaknesses:

The paper is very clearly written, and the experiments are robust, complete, and controlled. It is somewhat limited in scope, considering that Figure 1 and 5 could be supplementary data (setup of the system and negative data). However, the comparative approach and the controlled and well-known system allow the extraction of meaningful information in a field that has struggled to find specific anticancer approaches. In this sense, the authors contribute limited but highly valuable information.

Comments on revised version:

I reviewed the changes and response to criticism, and it seems to me that all has been reasonably addressed.

# Referee report: Reviewer #3 (Public review)

**DOI:** 10.7554/eLife.108812.3.sa2

Summary:

This manuscript by Shimizu et al., systematically analyzes cancer-associated mutations in the Negative Regulatory Region (NRR) of Drosophila Notch to reveal diverse regulatory mechanisms with implications for cancer modelling and therapy development. The study introduces cancer-associated mutations equivalent to human NOTCH1 mutations, covering a broad spectrum across the LNR and HD domains. By linking mutant-specific mechanistic diversity to differential signaling properties, the work directly informs targeted approaches for modulating Notch activity in cancer cells. These are an exciting set of observations from S2 cells, which should be taken up further for further assessment in any physiological implications.

Strengths:

This manuscript by Shimizu et al., systematically analyzes cancer-associated mutations in the Negative Regulatory Region (NRR) of Drosophila Notch to reveal diverse regulatory mechanisms with implications for cancer modelling and therapy development. The study introduces cancer-associated mutations equivalent to human NOTCH1 mutations, covering a broad spectrum across the LNR and HD domains. The authors use rigorous phenotypic assays to classify their functional outcomes. By leveraging the S2 cell-based assay platform, the work identifies mechanistic differences between mutations that disrupt the LNR-HD interface, core HD, and LNR surface domains, enhancing understanding of Notch regulation. The discovery that certain HD and LNR-HD interface mutations (e.g., R1626Q and E1705P) in Drosophila mirror the constitutive activation and synergy with PEST deletion seen in mammalian T-ALL is nice and provides a platform for future cancer modelling. Surface-exposed LNR-C mutations were shown to increase Notch protein stability and decrease turnover, suggesting a previously unappreciated regulatory layer distinct from canonical cleavage-exposure mechanisms. By linking mutant-specific mechanistic diversity to differential signaling properties, the work directly informs targeted approaches for modulating Notch activity in cancer cells.

Weaknesses:

This is an exciting set of observations, however the work is entirely cell line based, and is the primary weakness. I list my main specific concerns herewith:

(1) The analysis is confined to Drosophila S2 cells, which may not fully recapitulate tissue or organism-level regulatory complexity observed in vivo.

(2) And perhaps for this reason too, some Drosophila HD domain mutants accumulate in the secretory pathway and do not phenocopy human T-ALL mutations. Possibly due to limitations on physiological inputs that S2 cells cannot account for or species-specific differences such as the absence of S1 cleavage. Thus, the findings may not translate directly to understanding Notch 1 function in mammalian cancer models.

(3) Also, while the manuscript highlights mechanistic variety, the functional significance of these mutations for hematopoietic malignancies or developmental contexts in live animals remains untested. Thus even though the changes are evident in Notch signaling, any impact on blood cells or hematopoiesis leading to aberrant malignancies remains to be seen.

(4) Which hematopoietic cell type, progenitor or differentiating cells, would be most sensitive to this kind of altered Notch signaling also remains unclear.

# Author comment: Author response

**DOI:** 10.7554/eLife.108812.3.sa3

**Authors:** Hideyuki Shimizu, Martin Baron

**Affiliations:** University of Manchester Manchester United Kingdom; University of Manchester Manchester United Kingdom

The following is the authors’ response to the original reviews.

**Public Reviews:**

**Reviewer #1 (Public review):**

Summary:

In their paper, Shimizu and Baron describe the signaling potential of cancer gain-of-function Notch alleles using the Drosophila Notch transfected in S2 cells. These cells do not express Notch or the ligand Dl or Dx, which are all transfected. With this simple cellular system, the authors have previously shown that it is possible to measure Notch signaling levels by using a reporter for the 3 main types of signaling outputs, basal signaling, ligand-induced signaling and ligand-independent signaling regulated by deltex. The authors proceed to test 22 cancer mutations for the above-mentioned 3 outputs. The mutation is considered a cluster in the negative regulatory region (NRR) that is composed of 3 LNR repeats wrapping around the HD domain. This arrangement shields the S2 cleavage site that starts the activation reaction.

The main findings are:

(1) Figure 1: the cell system can recapture ectopic activation of 3 existing Drosophila alleles validated in vivo.

(2) Figure 2: Some of the HD mutants do show ectopic activation that is not induced by Dl or Dx, arguing that these mutations fully expose the S2 site. Some of the HD mutants do not show ectopic activation in this system, a fact that is suggested to be related to retention in the secretory pathway.

(3) Figure 3: Some of the LNR mutants do show ectopic activation that is induced by Dl or Dx, arguing that these might partially expose the S2 site.

(4) Figure 4-6: 3 sites of the LNR3 on the surface that are involved in receptor heterodimerisation, if mutated to A, are found to cause ectopic activation that is induced by Dl or Dx. This is not due to changes in their dimeriation ability, and these mutants are found to be expressed at a higher level than WT, possibly due to decreased levels of protein degradation.

Strengths and Weaknesses:

The paper is very clearly written, and the experiments are robust, complete, and controlled. It is somewhat limited in scope, considering that Figure 1 and 5 could be supplementary data (setup of the system and negative data). However, the comparative approach and the controlled and well-known system allow the extraction of meaningful information in a field that has struggled to find specific anticancer approaches. In this sense, the authors contribute limited but highly valuable information.

**Reviewer #2 (Public review):**

Summary:

This ambitious study introduced 22 mutations corresponding to amino acid substitution mutations known to induce cancer in human Notch1, located within the Negative Regulatory Region, into the Drosophila Notch gene. It comprehensively examined their effects on activity, intracellular transport, protein levels, and stability. The results revealed that the impact of amino acid substitutions within the Negative Regulatory Region can be grouped based on their location, differing between the Heterodimerization Domain and the Lin12/Notch Repeat. These findings provide important insights into elucidating the mechanisms by which amino acid substitution mutations in human Notch1 cause leukemia and cancer.

Strengths:

In this study, the authors successfully measured the activity of amino acid-substituted Notch with high precision by effectively leveraging the advantages of their previously established experimental system. Furthermore, they clearly demonstrated ligand-dependent and Deltex-dependent properties.

Weaknesses:

Amino acid substitution mutations exhibit interesting effects depending on their position, so interest naturally turns to the mechanisms generating these differences. Unfortunately, however, elucidating these mechanisms will require considerable time in the future. Therefore, it is reasonable to conclude that questions regarding the mechanism fall outside the scope of this paper.

We thank the editors and reviewers for their initial reviews and constructive suggestions. We have revised the manuscript with some additional data contained in two additional supplementary figures and by the inclusion of additional text.

**Reviewer #3 (Public review):**

While this is indeed an exciting set of observations, the work is entirely cell-line-based, and is the primary reason why this approach dampens the enthusiasm for the study. The analysis is confined to Drosophila S2 cells, which may not fully recapitulate tissue or organism-level regulatory complexity observed in vivo. Some Drosophila HD domain mutants accumulate in the secretory pathway and do not phenocopy human T-ALL mutations. Possibly due to limitations on physiological inputs that S2 cells cannot account for, or species-specific differences such as the absence of S1 cleavage.

Thus, the findings may not translate directly to understanding Notch 1 function in mammalian cancer models. While the manuscript highlights mechanistic variety, the functional significance of these mutations for hematopoietic malignancies or developmental contexts in live animals remains untested. Overall, the work does not yet provide evidence for altered Notch signaling that is physiologically relevant.

S2 cells are a standard cell culture model which have been extensively used for analysing Notch signalling mechanisms and by and large are found to recapitulate the mechanisms of Notch activation and its regulation in vivo. However, we agree that it will be desirable in future work to build on our current findings by generating Notch mutants in vivo in Drosophila as the in vivo context may introduce additional nuances in the behaviour of the mutants.This can be done by overexpressing cDNA constructs in particular tissues, or more physiologically by generating endogenous gene mutations using CRISPR/Cas9 based gene editing. However, the likely outcome of the latter approach is embryo lethality due to constitutive over-activation during development. Therefore, methods of genetic manipulation need to be applied which allow the final activating mutant form to be generated in somatic clones. We feel that this would be considerable amount of additional work and is out of scope for the current study, but we look forward to developing this approach in future work.

**Recommendations for the authors:**

**Reviewing Editor Comments:**

(a) Table 1: Explain the rationale for mapping non-conserved residues between human and fly Notch; consider adding an alignment or supplementary figure.

We have added a new Supplementary figure S2 showing an alignment of Notch sequences from different species to indicate the degree of conservation at the sites chosen for our mutagenesis study. Some locations were highly conserved and some locations less so. Both conserved and non-conserved residues were included to examine how structural perturbations at equivalent positions affect signalling activity, independent of sequence conservation. In addition to the new supplementary figure, we have changed the text in the Table 1 legend to clarify.

(b) Add or discuss data connecting LNR and HD mutant expression levels with stability and degradation mechanisms.

We have added additional text in the results section referring to Fig6A/B regarding the varying Notch protein levels between the different mutants. With regard to the slower degradation kinetics of certain LNR-C mutants in Fig6 E/F, we have also added a new supplementary figure S3 which shows that mutants from the LNR/HD interface do not behave similarly to the LNR-C mutants with respect to their degradation kinetics.

(c) Some mutants, especially those retained in the secretory pathway, are insufficiently characterized. The mechanism underlying their differential trafficking and stability remains underexplored.

We have added some extra text to the discussion section which explores the issue of secretory pathway retention of HD mutants in Drosophila cells further.

(2) Figure Legends:

(a) Figure 1A - Explain the ribbon vs. space-filling representation and color coding; include a definition of the Heterodimerization Domain.

We have added extra text to the Figure 1A legend

(b) Figure 2E - Clarify mutant selection; if possible, include additional examples for consistency.

We added extra text regarding selection of mutants for study into the legend of Figure 2

(c) Figure 3-4 - Explain logic for alanine substitutions; discuss difference at residue 1570 (P vs. A).

We added the following text to the result section. “Y1532 and Y1535 are not mutated in human cancers and therefore could not be assessed through patient-derived variants. Alanine substitution provides a controlled way to probe their contribution to NRR integrity and activation sensitivity by selectively removing their side-chain interactions while preserving overall fold.” We added extra text in the discussion section regarding the differences in the outcomes of the 1570 to A and P mutations.

(d) Figure 4 - Improve resolution and legibility.

We have replaced figure 4.

(e) Figure 6C - Correct residue numbering (1563, 1566).

Thank you for spotting this. This has been corrected.

(f) Figure 6F - Include control where protein levels do not increase.

A new supplementary figure S3 has been added which included this control data.

(3) Contextual and Conceptual Framing:

(a) Incorporate the limitations of the S2 system, and delineate which mechanistic insights are likely conserved versus those that may be species- or context-specific.

We have incorporated text to discuss S2 cell limitations.

(b) The study does not test functional consequences in hematopoietic or developmental contexts. Expand the discussion to emphasize how these cell-based findings could inform future in vivo studies or mammalian cancer modeling.
